# Supplementary material for: BLOC1S1 control of vacuolar organelle fidelity modulates TH2 cell immunity and allergy susceptibility
Source: bioRxiv. 2024 Mar 27:2024.03.21.586144. Preprint. [Version 1] doi: 10.1101/2024.03.21.586144 (PMC11722528; doi:10.1101/2024.03.21.586144)
Supplement: Supplement 1 [file media-1.pdf]

Supplementary Materials

TITLE

BLOC1S1 control of vacuolar organelle fidelity modulates T_H2 cell immunity and allergy susceptibility.

AUTHORS

Rahul Sharma[1], Kaiyuan Wu[2], Kim Han[1], Anna Chiara Russo[1], Pradeep K. Dagur[3], Christian Combs[4], Michael N. Sack[1]

Suppl. Figure 1. BLOC1S1 depleted $CD4^+$ T cells preferentially augments T_H2 immune response.

Suppl. Figure 2. Increased $Lamp1^+$ Cells in $BLOC1S1^{-/-}$ $CD4^+$ T-cells.

Suppl. Figure 3. STING inhibition and its siRNA KD reduces IFN-γ levels in CTRL and $BLOC1S1^{-/-}$ $CD4^+$ T-cells.

Suppl. Figure 4. TKO mice are more susceptible to drug induced dermatitis than CTRL mice.

****Suppl. Table 1.** List of primers used for qRT-PCR analysis.

****Suppl. Table 2.** List of antibodies used for immunofluorescence and immunoblotting.

****Suppl. Table 3.** List of FACS antibodies used and panel design for FACS analysis.

****Suppl. Table 4.** List of reagents used.

Supplementary Figure 1

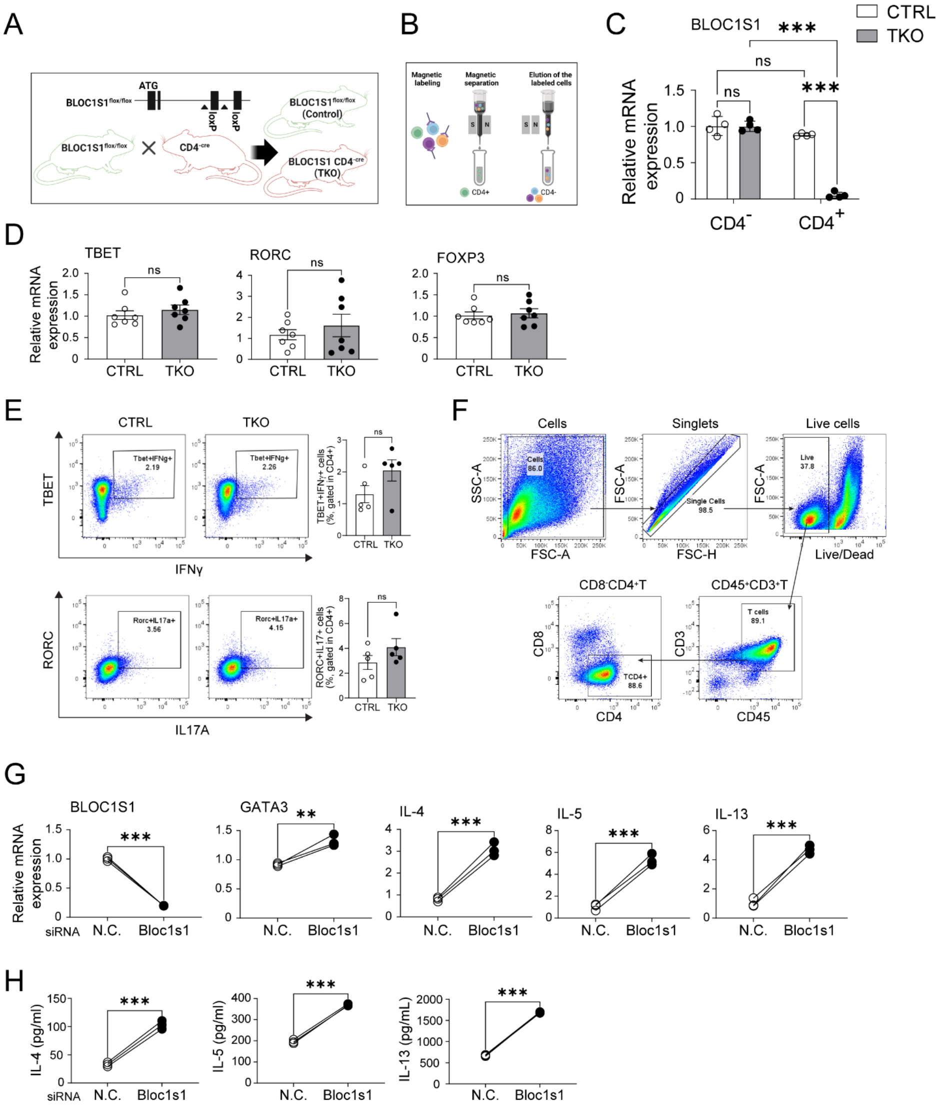

Figure S1. BLOC1S1 depleted CD4$^+$ T cells preferentially augments T$_H$2 immune response.

(A) Schematic representation of approach to generate CD4$^+$ cell specific BLOC1S1 knockout (TKO) mouse. **(B)** Schematic representation of approach to separate CD4$^+$ T cells from the residual splenic pool (CD4$^-$ cells). **(C)** qRT-PCR showing relative mRNA expression levels of BLOC1S1 in CD4$^+$ and CD4$^-$ cells (n=4 group). **(D)** qRT-PCR showing relative mRNA expression levels of TBET, RORC and FOXP3 in CD4$^+$ T cells (n=7 per group). **(E)** Representative flow-cytometric analysis of intracellular cytokines TBET$^+$IFN-γ$^+$ and RORC$^+$IL17$^+$ in CD4$^+$ T cells (n=5 per group). **(F)** Representative flow-cytometry gating profile of CD4$^+$ T cells for flow cytometry. **(G)** qRT-PCR showing relative mRNA expression levels of BLOC1S1, GATA3, IL-4, IL-5 and IL-13 in CD4$^+$ T cells isolated from blood of healthy individuals treated with either N.C. or siRNA (n=3 individuals). **(H)** IL-4, IL-5 and IL-13 cytokine release in activated CD4$^+$ T cells isolated from blood of healthy individuals (n=3 per group). Values represent mean ± SEM. *p<0.05, **p<0.01, ***p<0.001 vs. control mice using unpaired two-tailed student-t-test. FSC, forward scatter; SSC, side scatter.

Supplementary Figure 2

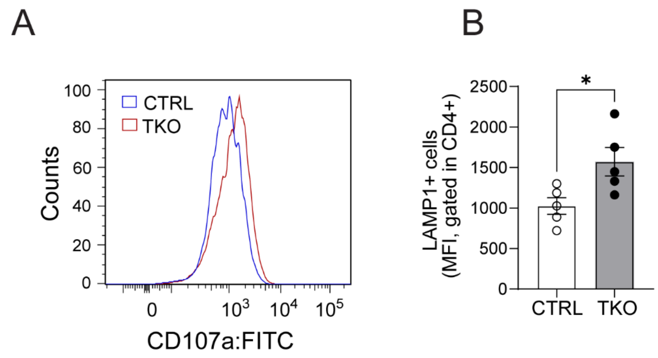

Figure S2. Increased Lamp1$^+$ Cells in BLOC1S1$^{-/-}$ CD4$^+$ T cells.

(A) Intracellular staining of CTRL and TKO mouse CD4$^+$ T cells with CD107a (Lamp-1). **(B)** Histogram of Lamp-1$^+$ in CD4$^+$ T cells (n=5 per group). Values represent mean +/- standard error of mean (SEM). *p<0.05, **p<0.01, ***p<0.001 vs. CTRL by using unpaired two-tailed student-t-test.

Commented [HKK([1]: It's too big? Please adjust it with same size of other figures

Supplementary Figure 3

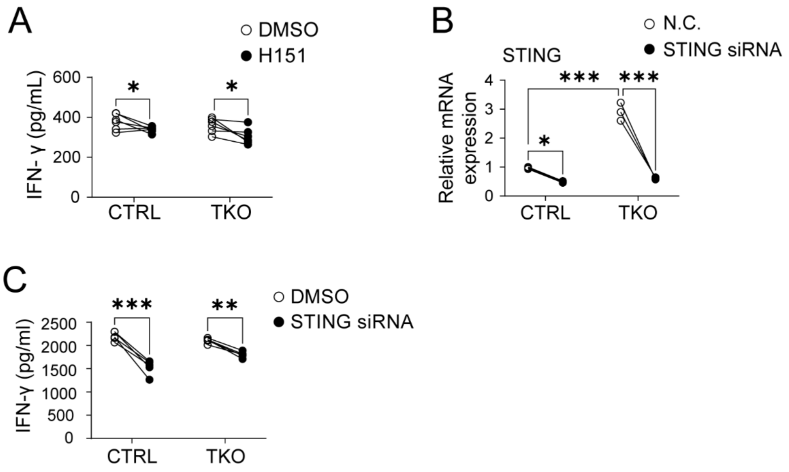

Figure S3. STING inhibition and its siRNA KD reduces IFN-γ levels in CTRL and BLOC1S1$^{-/-}$ CD4$^+$ T cells.

(A) IFN-γ cytokine release in CTRL and TKO CD4$^+$ T cells treated with either DMSO or H151 (500 nM) (n=6 per group). **(B)** qRT-PCR showing relative mRNA expression levels of STING in CD$^+$ T cells of CTRL and TKO treated with either N.C. or STING siRNA (n=3 per group). **(C)** IFN-γ cytokine release in CTRL and TKO CD4$^+$ T cells treated with either N.C. or STING siRNA (n=6 per group). Values represent mean $\pm$ SEM. *p<0.05, **p<0.01, ***p<0.001 vs control mice by two-way ANOVA followed by the Tukey's post hoc test or unpaired two-tailed student-t-test.

Supplementary Figure 4

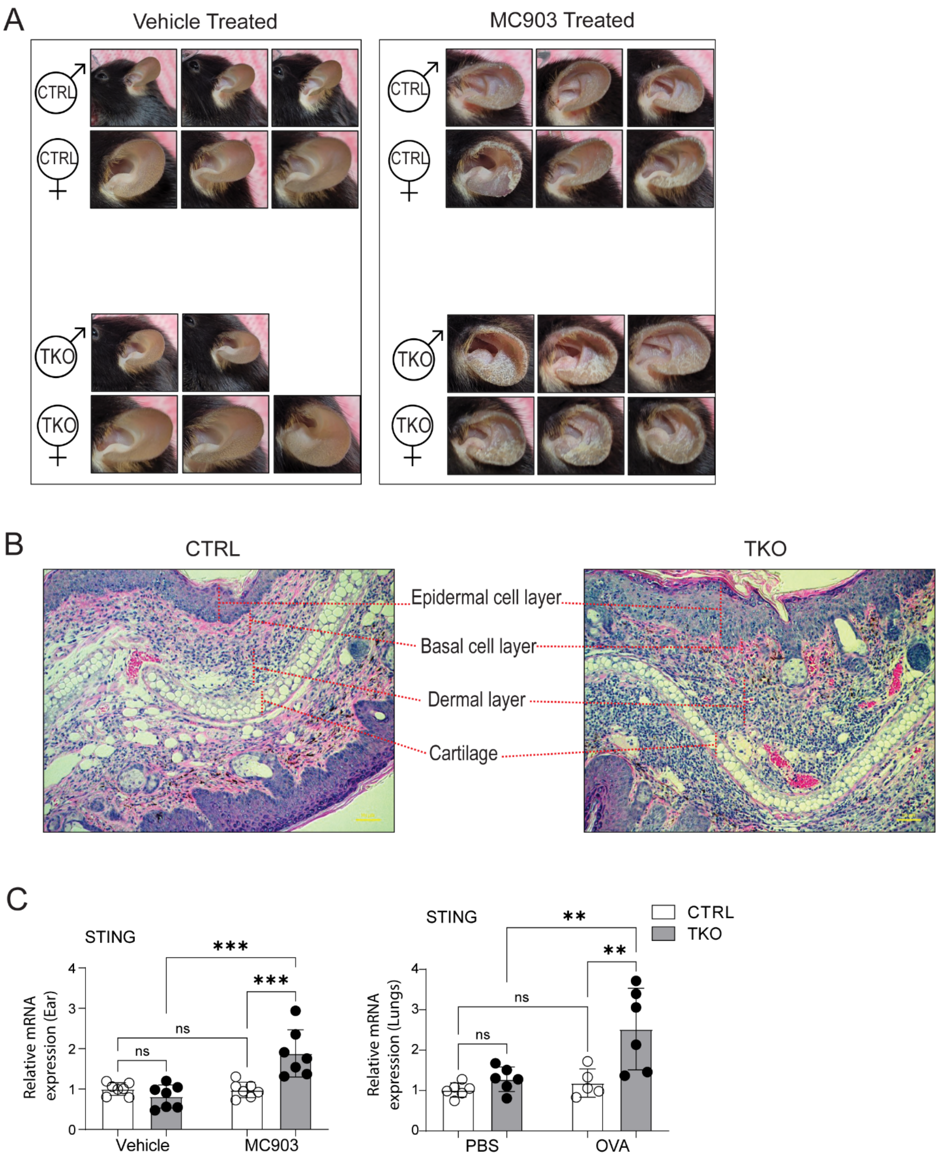

100

101

Figure S4. TKO mice are more susceptible to drug induced dermatitis than CTRL mice.

(A) Gross appearance of ears of mice treated with either ethanol or MC903 at day 12 in both sexes. **(B)** Relative H&E staining of MC903 treated CTRL and TKO mice ear sections at day 12. **(C)** qRT-PCR showing relative mRNA expression levels of STING in MC903 treated ear and OVA treated lungs (n=5-7 per group). Values represent mean $\pm$ SEM. *p<0.05, **p<0.01, ***p<0.001 vs. control mice by two-way ANOVA followed by the Tukey's post hoc test or unpaired two-tailed student-t-test.

Supplementary Table 1.

Sequence of primers used for qRT-PCR studies.

**Name**	**Source**	**Oligonucleotides**
IL-4 (Mouse)	Integrated DNA Technologies	F: CAAACGTCCTCACAGCAACG<br>R: TGCAGCTCCATGAGAACACTAG
IL-5 (Mouse)	Integrated DNA Technologies	F: AGCAATGAGACGATGAGGCTTC<br>R: CCCACGGACAGTTTGATTCTTCAG
IL-13 (Mouse)	Integrated DNA Technologies	F: AAGATCTGTGTCTCTCCCTCTGAC<br>R: ATACCATGCTGCCGTTGCAC
RNR2 (Mouse)	Integrated DNA Technologies	F: CTAGAAACCCCGAAACCAAA<br>R: CCAGCTATCACCAAGCTCGT
D-Loop (Mouse)	Integrated DNA Technologies	F: AATCTACCATCCTCCGTGAAACC<br>R: TCAGTTTAGCTACCCCCAAGTTTAA
TERT (Mouse)	Integrated DNA Technologies	F: CTAGCTCATGTGTCAAGACCCTCTT<br>R: GCCAGCACGTTTCTCTCGTT
BLOC1S1 (Mouse)	Integrated DNA Technologies	F: TCCCGCCTGCTCAAAGAAC<br>R: GAGGTGATCCACCAACGCTT
FoxP3 (Mouse)	Integrated DNA Technologies	F: CACCCAGGAAAGACAGCAACC<br>R: GCAAGAGCTCTTGTCCATTGA
T-bet (Mouse)	Integrated DNA Technologies	F: TCAACCAGCACCAGAGAGAG<br>R: AAACATCCTGTAATGGCTTGTG
Rorc (Mouse) QuantiTech Primer	Qiagen	Cat. QT00197722
18S (Mouse) QuantiTech Primer	Qiagen	Cat. QT02448075

Commented [HKK([2]: Table line space and style looks different
As well as please use same style of table

16S (Mouse)	Integrated DNA Technologies	F: CTAGAAACCCCGAAACCAAA<br>R: TCAGTTTAGCTACCCCCAAGTTTAA
IL-4 (Human) QuantiTech Primer	Qiagen	Cat. QT00012565
IL-5 (Human) QuantiTech Primer	Qiagen	Cat. QT00001435
IL-13 (human)	Qiagen	Cat. QT00000511
GATA3 (Human)	Integrated DNA Technologies	F: GAACCGGCCCCTCATTAAG<br>R: ATTTTTCGGTTTCTGGTCTGGAT
BLOC1S1 (Human) QuantiTech Primer	Qiagen	Cat. QT0016002
18S (Human) QuantiTech Primer	Qiagen	Cat. QT00199367

Supplementary Table 2.

Antibodies used for immunoblotting and Immunofluorescence			
**Antibody**	**Catalog #**	**Working dilution**	**Source**
Ki67 (D3B5)	9129S	1:400 (IF)	Cell Signaling
P-IKB-alpha (Ser32) (14D4)	2859S	1:1000 (IB)	Cell Signaling
IKB-alpha	9242S	1:1000 (IB)	Cell Signaling
P-NFkB P65 (Ser536) (93H1)	3033S	1:1000)IB)	Cell Signaling
NFkB P65 (D14E12)	8242S	1:1000 (IB)	Cell Signaling
P-STAT6 (Tyr641) (D8S9Y)	56554S	1:1000 (IB)	Cell Signaling
STAT6 (D3H4)	5397S	1:1000 (IB)	Cell Signaling
GATA3 (D13C9)	5852S	1:1000 (IB)	Cell Signaling
β-actin (8H10D10)	3700S	1:1000 (IB)	Cell Signaling
Total Oxphos	Ab110413	1:1000 (IB)	Abcam
cGAS (D3080)	31659S	1:1000 (IB)	Cell Signaling
P-STING (Ser365) (D8F4W)	72971S	1:1000 (IB)	Cell Signaling
STING (D2P2F)	13647S	1:1000 (IB), 1:200 (IF)	Cell Signaling
P-TBK1 (Ser172) (D52C2)	5483S	1:1000 (IB)	Cell Signaling
TBK1 (E813G)	38066S	1:1000 (IB)	Cell Signaling
Tom20 (F-10)	SC-17764	1:1000 (IB), 1:200 (IF)	Santa Cruz
VDAC (D73D12)	4661S	1:1000 (IB)	Cell Signaling
Lamp1 (ab208943)	Ab208943	1:1000 (IB), 1:200 (IF)	Abcam
LC3 AB (D3U4C)	12741S	1:1000 (IB), 1:200 (IF)	Cell Signaling
dsDNA (HYB331-01)	SC-58749	1:200 (IF)	Santa Cruz
Lamp1 (H4A3)	SC-20011	1:1000 (IB), 1:200 (IF)	Santa Cruz

Supplementary Table 3.

Antibody panel design for flow cytometry.

Target	Version	Catalog#	Vendor
CD4	BUV 395	563790	BD
CD3	BUV496	741117	BD
IFN-g	BUV737	612769	BD
CD45	BUV805	568336	BD
LIVE_DEAD	BV421	423114	BIOLEGEND
TNF-a	BV510	506339	BIOLEGEND
CD8	BV605	563152	BD
RORGT	BV650	564722	BD
IL-4	BV711	504133	BIOLEGEND
IL-17A	BV786	564171	BD
CD107A	FITC	121606	BIOLEGEND
CD27	PERCPCY5.5	563603	BD
IL-13	PE	568551	BD
TBET	PECF594	562467	BD
MHC-II	PECY7	107630	BIOLEGEND
FOXP3	APC	567462	BD
GATA3	APC-700	567633	BD
CD44	APCCY7	103028	BIOLEGEND
	FOXP3 BUFFER	00-5523-00	E BIOSCIENCE
	leukocyte activation cocktail	550583	BD

Supplementary Table 4.

List of reagents.

Reagent	Catalog number	Source
CyQuant Cell Proliferation Assay	C7026	Invitrogen
Pierce BCA Protein Assay	23227	Thermo Scientific
Human IL-4 Duoset ELISA Kit	DY204	R&D Systems
Human IL-5 Duoset ELISA Kit	DY205	R&D Systems
Human IL-13 Duoset ELISA Kit	DY213	R&D Systems
Mouse IFN-gamma Duoset ELISA Kit	DY485	R&D Systems
Mouse TNF-alpha Duoset ELISA Kit	DY410	R&D Systems
Mouse IL-4 Duoset ELISA Kit	DY404	R&D Systems
Mouse IL-5 Duoset ELISA Kit	DY405	R&D Systems
Mouse IL-13 Duoset ELISA Kit	DY413	R&D Systems
Mouse IL-10 Duoset ELISA Kit	DY417	R&D Systems
Mouse IL-17 Duoset ELISA Kit	DY421	R&D Systems
Lymphocyte Separation Medium	0850494	MP Biomedicals

Human CD4+ T Cell Isolation Kit	130-096-533	Miltenyi Biotec
NucleoSpin RNA Kit	740955	Macherey-Nagel
First-strand Synthesis SuperMix	11752250	Invitrogen
FastStart Essential DNA Green Master Mix	06924204001	Roche Life Science
Accell siRNA delivery media	B-005000-100	Horizon Discovery
H151	HY-112693	MedChemExpress
JSH-23	HY-13982	MedChemExpress
Rapamycin	HY-10219	MedChemExpress
SMARTpool: Accell BLOC1S1	E-012580-00-0020	Dharmacon
SMARTpool: Accell Tmem173	E-055528-00-0020	Dharmacon
CD3 (Mouse)	100339	Biolegend
CD28 (Mouse)	102116	Biolegend
